# Sperm immobilization test and quantitative sperm immobilization test using frozen‐thawed sperm preparation applied with computer‐aided sperm analysis

**DOI:** 10.1002/rmb2.12387

**Published:** 2021-05-18

**Authors:** Yu Wakimoto, Atsushi Fukui, Teruhito Kojima, Goh Wakimoto, Naoya Okamura, Hidetake Kamei, Yukiko Sugiyama, Toru Kato, Akiko Hasegawa, Hiroaki Shibahara

**Affiliations:** ^1^ Department of Obstetrics and Gynecology Hyogo College of Medicine Nishinomiya Japan; ^2^ Wakimoto Ob&Gyn Clinic Osaka Japan

**Keywords:** antisperm antibody, computer‐aided sperm analysis, frozen‐thawed sperm, sperm immobilization test, sperm‐immobilizing antibody

## Abstract

**Purpose:**

In a previous study, a new method was described using the sperm immobilization test (SIT) with computer‐aided sperm analysis (CASA). However, obtaining high‐quality sperm as needed was a known issue. Here, we compared the results of using frozen‐thawed sperm and fresh sperm for the SIT using the CASA method.

**Methods:**

For the frozen‐thawed preparation, 500 μL of condensed semen and 500 μL of Sperm Freeze were mixed in a cryovial and cryopreserved in liquid nitrogen. Density gradient centrifugation was used for the collection of motile sperm in both the fresh and frozen‐thawed sperm preparations. A total of 50 serum samples were prepared for both the fresh and frozen‐thawed sperm with each sample tested containing 10 μL of serum, 1 μL of either fresh or frozen motile sperm suspension, and 2 μL of complement. Sperm motilities were measured using CASA after a 1‐hour incubation period for both fresh and frozen‐thawed sperm.

**Results:**

Both fresh and frozen‐thawed sperm reacted similarly when exposed to serum containing sperm‐immobilizing antibodies asserting the use of frozen‐thawed sperm for the diagnosis of immunological infertility.

**Conclusion:**

These results suggest the possibility of using cryopreserved sperm for the SIT when fresh sperm is unavailable.

## 1 INTRODUCTION

Antisperm antibodies (ASAs) are associated with both male and female subfertility.^1^, ^2^, ^3^ Sperm are foreign bodies for women and upon exposure, are sometimes recognized as antigens, which can cause an immunological reaction in the woman.^4^, ^5^ This response could lead to the production of ASAs as allogeneic antibodies that can attenuate the sperm's effectiveness.^1^ In men, ASAs could be produced as sperm surface autoantibodies due to an imbalance or dysregulation of the control mechanisms by means of an anatomic, physiologic, or immunological barrier. In this case, sperm motility is also impaired.^2^, ^3^

The sperm‐immobilizing (SI) antibodies in female serum, which is an ASAs, are secreted into the cervical mucus, follicle fluid, and fallopian tube. SI antibodies prevent the sperm from penetrating progressively into the cervical mucus and do not allow sperm to pass into the uterus.^6^ SI antibodies are also known to inhibit fertilization even if sperm are able to enter the uterus through the cervix and reach the fallopian tubes.^7^, ^8^ The sperm immobilization test (SIT) and quantitative SIT have been developed as an useful tool for detecting SI antibodies and determining the SI antibody titers (SI_50_: 50% sperm immobilization unit), respectively.^9^, ^10^ These tests have been traditionally calculated by eye estimation using a microscope to determine the proportion of motile sperm, after the number of motile and immotile sperm were counted manually by a highly trained laboratory embryologist.^9^ Recently, we outlined a new method for SIT and quantitative SIT using computer‐aided sperm analysis (CASA), which aimed to make the SIT and quantitative SIT more convenient and objective.^11^ However, in our previous study describing the new method using CASA, we only used fresh sperm when validating its effectiveness. In a practical setting, obtaining high‐quality fresh sperm from a donor is not always readily available. Therefore, in this study, we compared frozen‐thawed sperm with fresh sperm by using the new CASA method for SIT and quantitative SIT.

## 2 MATERIALS AND METHODS

### 2.1 Subjects

The study has been approved by the ethical committee (Hyogo College of Medicine College Hospital, Nishinomiya, Japan. No. 2865).

#### 2.1.1 Test serum

Fifty serum samples were collected from Japanese infertile women who received infertility treatment in our hospital from the year 2000 to 2020 and stored at −80°C. An opt‐out consent method was provided. The serum samples were prepared by centrifugation at 1500 *g* for 5 minutes, and inactivation of endogenous complement activities was treated by heating the sera at 56°C for 30 minutes.

#### 2.1.2 Complement

The complement (Low‐Tox guinea pig complement) was purchased from CADARLANE Lab. Lit. In some experiments, inactivation of the complement was treated at 56°C for 30 minutes to obtain a negative control.

#### 2.1.3 Sperm suspension

##### Sperm samples

Sixteen freshly ejaculated normal semen samples from five healthy men were obtained by masturbation from volunteers under informed consent. The motility assay was evaluated using CASA. Sperm samples with motility above 68% were included in this study. The mean ± SD of the sperm concentration and sperm motility was 88.4 ± 55.9 × 10^6^/mL (range: 34.3‐215.9) and 83.5 ± 7.3% (range: 68.1‐95.3), respectively. In both the fresh and the frozen‐thawed sperm, motile sperm for experimentation was collected using density gradient centrifugation. Sperm samples at a concentration of 100 × 10^6^/mL were prepared for experimentation.

##### Sperm cryopreservation

After liquefaction, the fresh semen samples were condensed by centrifugation at 540*g*  for 5 minutes. Subsequently, 500 μL of condensed semen and 500 μL of Sperm Freeze^®^ (Kitazato Co.) were mixed well in a cryovial. Subsequently, the cryovial with the mixture was frozen in liquid nitrogen (LN_2_) vapor for 5 minutes and submerged into LN_2_ for cryopreservation. Afterward, the cryovial was immediately transferred to a storage tank with the LN_2_.

##### Sperm thawing

For thawing, the cryovial was taken out of the storage tank with the LN_2_. After warming for 1 minute at room temperature, the frozen sperm in the cryovials were thawed by placing them in a warming solution at 37°C for 5 minutes.

### 2.2 Sperm immobilization test

Two wells in one Terasaki microplate were used for the SIT. Mixtures of 10 μL of serum, 1 μL of sperm suspension, and 2 μL of complement (one well contains the activated complement and another contains the inactive complement) were mixed in each of the two wells of the Terasaki microplate, and the mixtures were immediately applied to the chamber slides of 12‐µm thickness (LEJA) with the active complement on one side, and the inactive complement on the other. The mixtures were then incubated for 1 hour at room temperature. The same steps were followed using both the fresh and the frozen‐thawed sperm. Sperm motilities were measured using CASA (Ditect Co.) after the 1‐hour incubation for both fresh and frozen‐thawed sperm. The percentages of motile sperm with active and inactivated complement were calculated for T% and C%. C/T was designated as the sperm immobilization value (SIV). A SIV‐positive result for SI antibodies was defined as a value of 2 or more.

### 2.3 Quantitative sperm immobilization test

**FIGURE 1:**
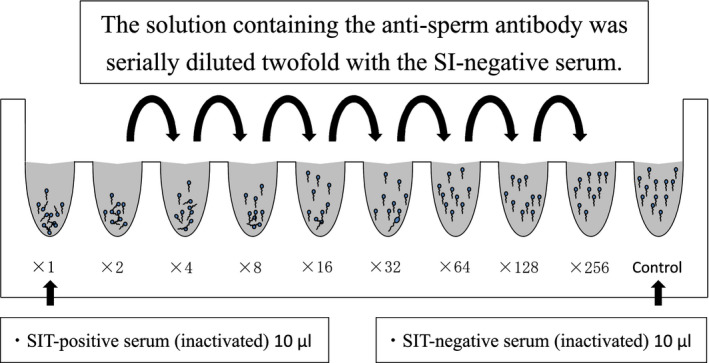
Procedure for the quantitative SIT. Ten wells in one Terasaki microplate were used. The 10 μL of heated‐inactivated SIT‐positive serum was added to the well of the ×1 and ×2. Subsequently, 10 μL of heated‐inactivated SIT‐negative serum was added to the well of the ×4, ×8, ×16, ×32, ×64, ×128, ×256, and control. Subsequently, after 10 μL of the heated‐inactivated SIT‐positive serum was mixed to well ×2, the solution of the well of the ×4 was diluted twofold with 10 μL of the solution of the well of the ×2. Similarly, the solution containing the antisperm antibody was serially diluted twofold with the SI‐negative serum until the well of the ×256

Ten wells in one Terasaki microplate were used for quantitative SIT. Figure 1 shows that 10 μL of heat‐inactivated SIT‐positive serum was added to the well of the ×1 and ×2. Subsequently, 10 μL of the heat‐inactivated SIT‐negative serum was added to the well of the ×4, ×8, ×16, ×32, ×64, ×128, ×256, and control. Afterward, after 10 μL of heat‐inactivated SIT‐positive serum was mixed into the well of the ×2, the solution of the ×4 well was diluted twofold with 10 μL of the solution of the well of the ×2. Similarly, the solution containing the antisperm antibody was serially diluted twofold with the SIT‐positive serum diluted by SIT‐negative serum until the well of ×256. Subsequently, 1 μL of the sperm suspension (100 × 10^6^/mL) and 2 μL of complement were mixed into each well. The mixtures were immediately applied to chamber slides with the mixture with fresh sperm on one side and the mixture with frozen‐thawed sperm on the other and incubated for 1 hour at room temperature. Sperm motility was analyzed using CASA after 1‐hour incubation for both fresh and frozen‐thawed sperm.

Sperm motility in the SIT‐positive serum and each of the diluted SIT‐positive and control sera was determined using T% and C%, respectively. Sperm immobilization activity was defined as the value of (C − T)/C × 100. Sigmoid curve was obtained by plotting the value of the sperm immobilization activity against the serum dilution. The serum dilution with 50% sperm immobilization activity was defined as the quantitative titer of SI antibody (SI_50_), indicating that the dilution time needed to recover 50% sperm motilities is defined as SI_50_.

The mixtures of the fresh and the frozen‐thawed sperm were incubated simultaneously, and motile sperm in both mixtures were counted at the same time using the same serum sample and complement.

### 2.4 Statistical analysis

The correlation between the values of SI_50_ obtained by both fresh and frozen‐thawed sperm was analyzed using JMP Pro 14 software (SAS Institute Inc). The correlation analysis was performed using Pearson's correlation coefficient. The differences were considered significant with a probability value of <.05.

## 3 RESULTS

Table 1 shows the results of the SIT. The SIV between fresh and frozen sperm was compared. In the SIT‐negative category, the SIV of fresh sperm had a mean value of 1.02 ± 0.14 (range: 0.76‐1.58) and frozen‐thawed sperm had a mean value of 1.03 ± 0.14 (range: 0.88‐1.68); the correlation of SIV between fresh and frozen‐thawed samples was 0.73. In the SIT‐positive category, the SIV of fresh sperm had a mean value of 32.87 ± 31.38 (range: 2.5‐141) and frozen‐thawed sperm had a mean value of 43.74 ± 37.73 (range: 3.6‐143.9). The correlation of the SIV between fresh and frozen‐thawed samples was 0.15 (note: sample NO.31 was considered an outlier and not included in the analysis). The results of SIT were identical; 21 of 50 samples tested positive and 29 samples were negative for SI antibodies, based on both fresh and frozen‐thawed sperm.

**TABLE 1:** Comparison of the SIV between fresh and frozen sperm

SIT negative		
Sample	SIV	
No.	Fresh	Frozen
1	0.99	0.93
2	0.96	0.88
3	1.04	0.88
4	1.00	0.90
5	1.06	0.97
6	1.04	1.10
7	0.98	0.95
8	0.90	1.11
9	1.01	0.96
10	0.98	1.02
11	0.98	1.13
12	1.01	0.98
13	0.98	1.01
14	1.02	0.93
15	1.00	1.03
16	1.01	1.03
17	1.02	1.12
18	1.08	1.06
19	0.96	0.97
20	0.76	1.00
21	0.99	1.04
22	0.93	1.02
23	0.99	0.92
24	0.90	0.94
25	1.03	1.17
26	1.38	1.13
27	1.07	1.05
28	1.04	1.04
29	1.58	1.68

SIT positive		
Sample	SIV	
No.	Fresh	Frozen
30	141.0	60.2
31	∞	26.8
32	44.5	52.7
33	29.9	125.4
34	97.0	12.2
35	15.6	8.8
36	23.9	22.4
37	14.7	50.1
38	33.3	70.0
39	19.5	8.7
40	31.3	36.3
41	15.5	24.1
42	37.1	143.9
43	2.5	6.3
44	34.2	13.5
45	5.5	3.6
46	21.4	72.6
47	26.2	69.0
48	35.3	21.6
49	16.1	50.1
50	12.8	23.3

Note

Table 1 shows that the results were identical, and 21 of 50 samples tested were positive, and 29 samples were negative for sperm‐immobilizing (SI) antibodies based on both fresh and frozen‐thawed sperm.

Abbreviations: ∞, infinity; SIT, Sperm immobilization test; SIV, Sperm immobilization value.

**FIGURE 2:**
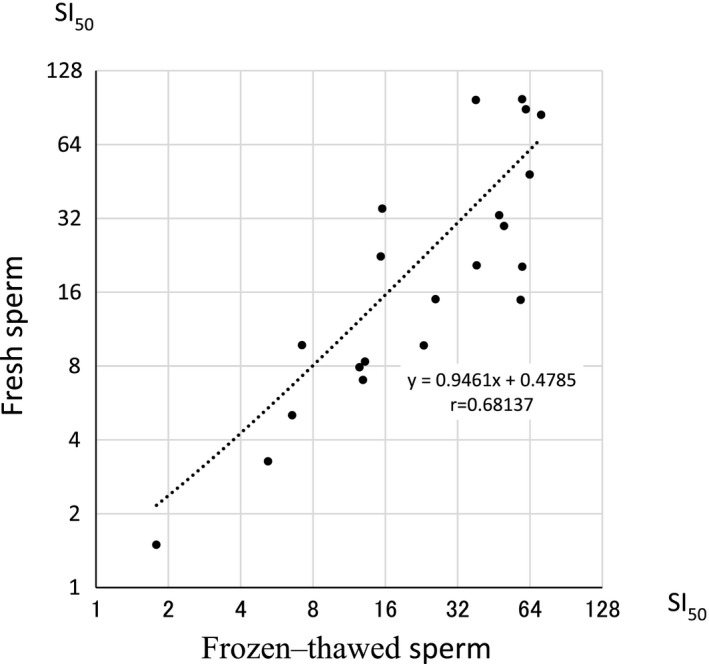
Values of SI_50_ obtained by both fresh and frozen‐thawed sperm. The values of SI_50_ obtained by both fresh and frozen‐thawed sperm were closely correlated with high co‐efficiency (*r* = .68, *P* < .01)

Figure 2 shows that the values of SI_50_ obtained by both fresh and frozen‐thawed sperm were closely correlated with high co‐efficiency (*r* = .68, *P* < .01).

## 4 DISCUSSION

Sperm are foreign antigens for women, and therefore, women might produce ASAs impairing sperm motility.^4^, ^5^ If women are exposed to sperm, ASAs might cause refractory infertility.^1^ Sperm function is adversely affected by ASAs, including inhibitory effects on sperm penetration through cervical mucus and inhibitory effects on fertilization even if sperm enters the uterine cavity and reaches the fallopian tubes.^6^, ^7^ However, not all ASAs interfere with sperm motility or fertilization at the same level.^12^ Women with a high titer of SI antibodies have difficulty in getting pregnant due to less responsiveness to infertility treatments by means of timed intercourse (TI) or intrauterine insemination (IUI).^13^ Once a woman is determined to have a high titer of SI antibodies, other means of fertilization should be recommended, such as in vitro fertilization (IVF).

Our results suggest that IVF is suitable as a treatment choice in patients with a high titer of SI antibodies. Patients with a high titer of SI antibodies have similar fertilization rate as patients with non‐SI antibodies if IVF is used.^14^ Therefore, it is important that the presence of SI antibodies is determined along with other standard tests for a clinical diagnosis of subfertility in women. It has been proposed that infertile patients should receive earlier examinations for SIT as a means of reducing treatment time so as not to consume their reproductive time using methods with a low chance of success such as TI or intrauterine IUI.^11^

However, routine screening of female serum for SI antibodies is performed in only few fertility clinics although SIT can be performed conveniently by obtaining the complement and sperm. One reason for this is that the screening of all patients undergoing assisted reproductive technology (ART) for sperm‐immobilizing antibodies in the patient's serum is ineffective because it may not change the management and prognosis of the patient undergoing ART.^14^ However, detecting the presence of ASA early in treatment regimens for infertile patients could reduce the amount of time wasted by treating subfertility with TI or IUI without knowing the presence of ASAs. Thus, SIT should be performed early during medical treatments for infertility in order to reduce treatment time. Another reason SIT is not routinely used in clinical diagnosis is that the test lacks standardization and objectivity because the method of counting sperm is done by eye estimation by a highly skilled embryologist.^9^ Although generally accurate enough, this method is still prone to human error. To manage this, we recently outlined a new method using CASA to make SIT evaluation more convenient and objective as compared to the traditional method.^11^ We found the assay results for detecting the SI antibodies and estimating the quantitative titer of SI antibodies to be significantly correlated between the traditional method of manually counting sperm by eye estimation by a highly skilled embryologist and the new method of counting sperm through the chamber slides using CASA.^11^ Moreover, this study provided evidence that a highly skilled embryologist is not necessarily required in order to perform this test. Another reason this test is not commonly done is that it is sometimes difficult to obtain high‐quality fresh semen from a donor when required. For this reason, in this study, we examined whether cryopreserved sperm could be used for SIT using the CASA method.

The antigens involved in agglutination responses are thought to be surface antigen.^15^ In case sperm antigens are recognized by a female patient's serum antisperm antibodies, this could affect the frozen‐thawing process. The frozen‐thawing process might affect the measurement of the SIT. Freezing and thawing have been reported to cause substantial protein changes.^16^ The study conducted by Alexander et al^15^ using the indirect immunofluorescence method demonstrated that the cryogenic freezing process caused the change of antigenicity of human sperm. They compared frozen‐thawed sperm with the fresh sperm using indirect immunofluorescence tests for antisperm antibodies and showed that the frozen‐thawing process decreased the presence of certain surface antigens. Therefore, frozen‐thawing process is thought to result in removal of spermatozoa membrane antigens. However, in the study conducted by Phillip et al,^17^ the antigenicity involved in immobilization of frozen‐thawed sperm has been reported to be unchanged and remained intact during the frozen‐thawing process. Using SIT, the immunoperoxidase assay, and separation of proteins by gel electrophoreses, they found there was no difference in the membrane antigens before and after frozen‐thawing process.^12^ Our results showed a significant correlation between fresh and frozen‐thawed sperm when detecting the presence of ASAs, thus supporting the findings that frozen‐thawed sperm remain unchanged throughout the freezing and thawing process. Moreover, a significant correlation was found in SI_50_ titer between fresh and frozen‐thawed sperm. Taken together, these results indicate that SIT and quantitative SIT can be performed using frozen‐thawed sperm instead of using fresh sperm.

Cryopreservation of human spermatozoa is widely used for infertility therapy, the improvement of ART, and male fertility preservation before cancer therapy.^18^, ^19^ Ice crystallization during the cooling process causes increased toxicity and deleterious effects to the sperm plasma membrane covering the sperm head.^19^ As such, the process of thawing sperm after cryopreservation leads to a significant decrease in spermatozoa viability, which decreases the number of motile normal sperm.^20^ However, high‐quality sperm must be obtained for SIT, and therefore, motile spermatozoa were collected using a Percoll density gradient method for SIT in our study.^21^ Based on our findings, the possibility of using cryopreserved sperm for detecting SI antibodies using the CASA method when fresh sperm is unavailable is both practical and recommended for clinical practice. However, there are possible limitations when using this test clinically. In particular, it is important to acknowledge the comparative sensitivity of the test in relation to the availability of high‐quality sperm. High‐quality sperm with good motility (in our case, above 68%) may be required to see clinically relevant results. Also, finding similar quality sperm in an amount necessary for clinical testing could be difficult.

In conclusion, we found that the method using CASA with frozen‐thawed sperm enables SIT to be more a more convenient and practical test as a clinical indicator.

## DISCLOSURES

*Conflict of interest*: The authors declare no conflict of interest. *Human rights statements and informed consent*: The protocol for the research project was approved by the Hyogo College of Medicine Ethics Committee, Nishinomiya, Japan. All the procedures were followed in accordance with the ethical standards of the responsible committees on human experimentation (institutional and national) and with the Helsinki Declaration of 1964 and its later amendments. Informed consent was obtained from all the patients for being included in the study. *Animal studies*: This article does not contain any studies with animal participants performed by any of the authors.
